# High expression of *COPB2* predicts adverse outcomes: A potential therapeutic target for glioma

**DOI:** 10.1111/cns.13254

**Published:** 2019-11-11

**Authors:** Yan Zhou, Xuan Wang, Xing Huang, Xu‐Dong Li, Kai Cheng, Hao Yu, Yu‐Jie Zhou, Peng Lv, Xiao‐Bing Jiang

**Affiliations:** ^1^ Department of Neurosurgery Union Hospital Tongji Medical College Huazhong University of Science and Technology Wuhan China

**Keywords:** biomarker, *COPB2*, gene set enrichment analysis, gene set variation analysis, glioma

## Abstract

**Aims:**

To evaluate the clinical significance of coatomer protein complex subunit beta 2 (*COPB2*) in patients with glioma using a bioinformatics analysis.

**Methods:**

Oncomine, GEO, and The Cancer Genome Atlas databases were used to examine the *COPB2* transcript levels in glioma tissues. Gene expression profiles with clinical information from low‐grade glioma and glioblastoma (GBM) projects were analyzed for associations between *COPB2* expression and clinicopathologic characteristics. Kaplan‐Meier survival and Cox regression analyses were used for survival analysis. Gene set enrichment analysis (GSEA) was conducted to screen the pathways involved in *COPB2* expression. Gene set variation analysis (GSVA) and correlograms were performed to verify the correlations between *COPB2* and inflammatory responses. Canonical correlation analyses examined whether *COPB2*‐high patients have more infiltrating inflammatory and immune cells.

**Results:**

*COPB2* was highly expressed in gliomas and high *COPB2* expression correlated with shorter overall survival time and several poor clinical prognostic variables. GSEA indicated that some immune‐related pathways and other signaling pathways in cancer were associated with the *COPB2*‐high phenotype. The GSVA and canonical correlation analysis demonstrated that *COPB2* expression was closely linked to inflammatory and immune responses, and higher immune cell infiltration.

**Conclusions:**

*COPB2* may be a potential prognostic biomarker and an immunotherapeutic target for glioma.

## 1 INTRODUCTION

Gliomas are the most common malignant brain tumors with high recurrence and lethality rates.^1^ According to the classification of the World Health Organization (WHO), brain gliomas are categorized into four grades (I‐IV).^2^ Glioblastoma multiforme (GBM, grade IV) exhibits a malignant phenotype associated with high proliferative activity, potent invasive ability, and vascular formation with a median survival rate of no more than 15 months postdiagnosis.^3^ With the development of biomedical techniques, several biomarkers and molecular classifications of glioma have been established. However, effective and reliable biomarkers that could predict poor prognosis and direct treatment strategies are rare. Thus, the identification of efficient neuropathological biomarkers and therapeutic targets is urgently required.

The coatomer protein complex subunit beta 2 (*COPB2*), also known as beta prime‐COP or beta‐Cop, is one of the seven subunits that form coatomer complex I.^4^ *COPB2* serves as a mediator in the process of protein synthesis which transports proteins from the endoplasmic reticulum (ER) to the Golgi apparatus.^5^ Recently, *COPB2* has been viewed as a new oncogene in many cancer types. Bhandari et al^6^ reported that the upregulation of *COPB2* in breast cancer is associated with age and lymph node metastasis in a validated cohort and promotes tumor cell proliferation and invasion. Further, a recent study demonstrated that *COPB2* could be a potential target gene in prostate cancer.^5^ Loss of function experiments demonstrated that *COPB2* downregulation arrests the cell cycle at G1 and G2 phases and induces cell apoptosis. Pu et al^7^ reported that *COPB2* upregulation in lung adenocarcinoma cell lines facilitates cell growth and tumorigenesis via upregulating *YAP1* expression. These previous findings indicated that *COPB2* might play a critical role in the development of cancer. Yet, the clinical significance of *COPB2* in glioma remains unclear.

Thus, this research aimed to reveal the association between *COPB2* and glioma and explore the potential prognostic value of *COPB2* in patients with glioma based on The Cancer Genome Atlas (TCGA), Oncomine, and the Gene Expression Omnibus (GEO) databases. The results indicated that *COPB2* was significantly overexpressed in glioma tissues compared with nontumor tissues and that high *COPB2* expression was correlated with higher WHO grade, shorter overall survival (OS) time, and several poor clinical prognostic variables. Gene set enrichment analysis (GSEA) showed that some immune‐related pathways and other signaling pathways in cancer were associated with the *COPB2* high expression phenotype, shedding light on the molecular mechanisms underlying the onset and progression of glioma. Gene set variation analysis (GSVA) and canonical correlation analysis demonstrated *COPB2* expression was closely linked to a higher infiltration of immune cells, as well as inflammatory and immune responses.

## 2 METHODS

### 2.1 Public database and bioinformatics analysis

The transcript level of *COPB2* in different cancers was ascertained by the Oncomine database (https://www.oncomine.org/resource/main.html),^8^ with a threshold set as such—top gene rank 10%, fold change >2, and *P*‐value <1E‐4. The microarray data of patients with glioma were downloaded from the GEO (https://www.ncbi.nlm.nih.gov/geo)^9^ public database under accession number http://www.ncbi.nlm.nih.gov/geo/query/acc.cgi?acc=GSE16011.^10^ Gene expression profile data containing clinical information from low‐grade glioma and GBM projects (HTSeq‐FPKM) were obtained from TCGA database (http://cancergenome.nih.gov/).^11^ The data from TCGA were further analyzed for associations between *COPB2* expression and clinicopathologic characteristics in glioma.

### 2.2 Gene set enrichment analysis and gene set variation analysis

To investigate the potential mechanisms underlying the interaction of *COPB2* expression on glioma progression, a GSEA^12^ was conducted to screen out whether some biological pathways showed statistically significant differences between high and low *COPB2* expression groups. For each analysis, gene set permutations were implemented 1000 times. Gene sets with a false discovery rate (FDR) <0.05 and normal *P*‐value <.05 were viewed as significantly enriched. Moreover, GSVA^13^ was performed to transform gene expression values into scores for inflammatory response metagene sets, followed by the application of correlograms to further verify correlations between *COPB2* and these metagenes.

### 2.3 Statistical analysis

The statistical analyses were performed utilizing R software v3.5.1. Descriptive statistics were used to summarize the molecular and clinical characteristics of patients in the TCGA database. To analyze potential relationships between *COPB2* and clinicopathologic features, Mann‐Whitney *U* and logistic regression tests were used. The Kaplan‐Meier method and Cox regression analyses were used to compare the impact of *COPB2* expression on the OS of TCGA patients alongside with other clinical variables. The remaining correlations between *COPB2* expression and inflammatory and immune cell types were detected by using canonical correlation analysis in GraphPad Prism 7 and SPSS 25.0. In all statistical analyses conducted, a *P*‐value <.05 was viewed as statistically significant.

## 3 RESULTS

### 3.1 Glioma *COPB2* transcript levels in different databases

**Figure 1:**
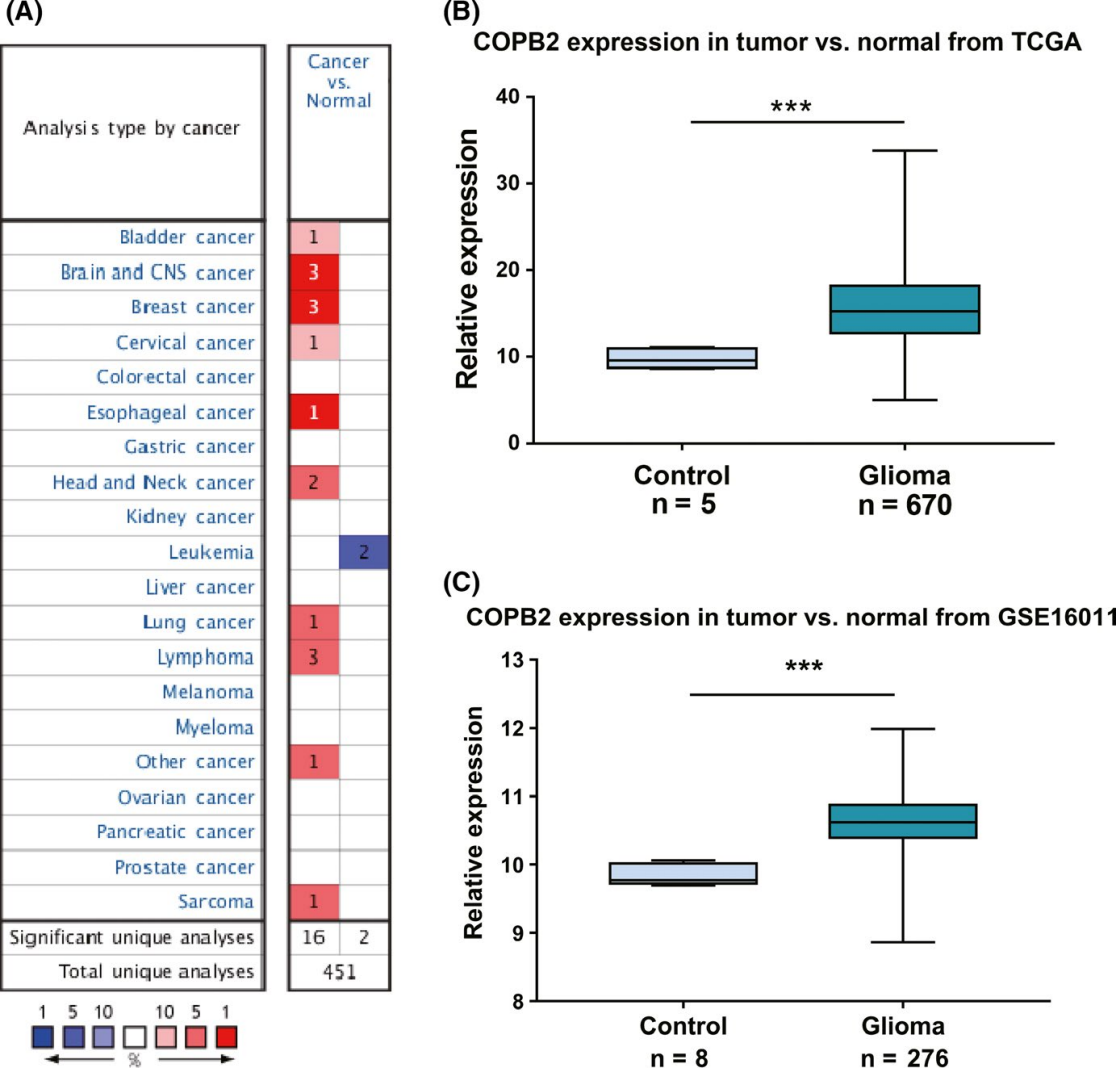
A, COPB2’ expression level in cancers in Oncomine Database: the left box in red indicated the number of datasets with COPB2 hyperexpression and the right box in blue indicated the number of datasets with COPB2 hypo expression after comparing cancerous and normal tissues. B, C, TCGA cohort and GSE16011 dataset from GEO support the findings that indicate COPB2 upregulation in glioma

Firstly, the transcript levels of *COPB2* in different cancers were analyzed. The Oncomine database (one of the main functions of which is gene expression differential analysis) was used to explore the expression of *COPB2* mRNA in different cancers (Figure 1A), and 189 datasets, including 33 144 samples, were included. Relative to normal clinical specimens, *COPB2* indicated significant hyper‐expression in bladder, brain and central nervous system, breast, esophageal, head and neck, lung, lymphoma, sarcoma, and other cancers, but hypo‐expressed in leukemia (Figure 1A), suggesting that the high expression of *COPB2* is common in various types of cancer. The detailed expression profile was summarized in Table S1.

In glioma, 675 glioma patients with *COPB2* expression profile data were obtained from TCGA *COPB2* is significantly upregulated in tumor tissues relative to nontumor tissues (Figure 1B, *P* < .001). In addition, we also used the http://www.ncbi.nlm.nih.gov/geo/query/acc.cgi?acc=GSE16011 dataset from GEO database for the purpose of validation (Figure 1C, *P* < .001). The results indicated increased transcript levels of *COPB2* in glioma.

### 3.2 TCGA glioma patient characteristics

As the TCGA database contains sufficient glioma samples, we only selected this database for further analysis of the association between gene and clinical characteristics. A total of 1114 cases (Table 1) with both gene expression and clinical data were available for 194 astrocytomas, 191 oligodendrogliomas, 130 oligoastrocytomas, 596 GBMs, and three cases that lacked histological information. The median age of patients was 52 years old, and there were 651 men and 460 women. The study cohort included 249 grade II, 265 grade III, and 596 grade IV cases, but unfortunately did not include any grade I cases. Only 125 cases were tested for the *IDH1* mutation—8.17% (n = 91) were classified as a mutated type while 3.05% (n = 34) were not. With respect to KPS (Karnofsky Performance Score), 52.4% (n = 584) patients scored ≥80 points and 13.6% (n = 151) were <80 points. There were 209 tumor‐free (18.8%) and 783 tumor (70.3%) patients. A family history of cancer was present in 132 cases (11.9%). In respect to ethnicity, 4.04% (n = 45) were Hispanic or Latino, while the majority of cases (84.3%, n = 939) were not Hispanic or Latino. Finally, 51.7% (n = 570) patients were alive at last follow‐up contact and 48.4% (n = 539) were dead, while five patients lost contact.

**Table 1:** The Cancer Genome Atlas glioma patient characteristics

Clinical characteristics		No. of patients	Percentage (%)
Age (y)	9‐89	Median 52	
Sex	Male	651	58.4
	Female	460	41.3
	Missing	3	0.270
Vital status	Alive	570	51.2
	Dead	539	48.4
	Missing	5	0.450
WHO grade	G2	249	22.4
	G3	265	23.8
	G4	596	53.5
	Missing	4	3.60
Histology	Astrocytoma	194	17.4
	Oligodendroglioma	191	17.2
	Oligoastrocytoma	130	11.7
	Glioblastoma	596	53.5
	Missing	3	0.270
KPS	<80	151	13.6
	≥80	584	52.4
	Missing	379	34.0
Tumor status	Tumor‐free	209	18.8
	With tumor	783	70.3
	Missing	122	11.0
*IDH1* mutation	Yes	91	8.17
	No	34	3.05
	Missing	989	88.8
Family history of cancer	Yes	132	11.9
	No	210	18.9
	Missing	772	69.3
Ethnicity	Hispanic or Latino	45	4.04
	Not Hispanic or Latino	939	84.3
	Missing	130	11.7

### 3.3 Association with *COPB2* expression and clinicopathologic features

**Figure 2:**
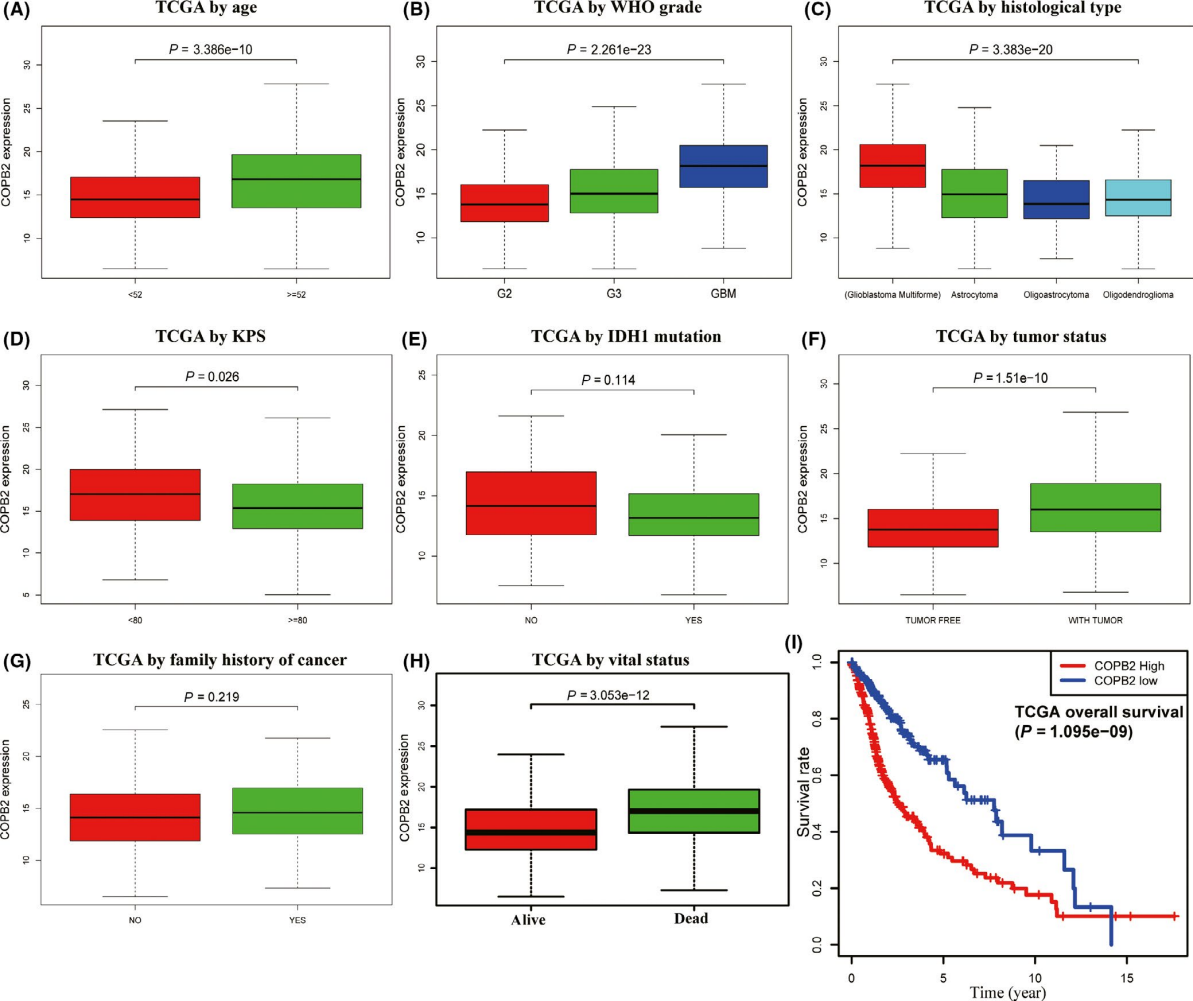
Associations between COPB2 expression and clinicopathologic variables in TCGA cohort, including (A): age, (B): WHO Grade, (C): histological type, (D): KPS, (E): IDH1 mutation, (F): tumor status, (G): family history of cancer, (H): vital status, and (I): influence of COPB2 expression on overall survival of glioma patients in TCGA cohort

To explore the expression pattern of *COPB2* in gliomas, mRNA expression profiles from the TCGA database were obtained and analyzed. As shown in Figure 2(A‐H), increased expression of *COPB2* correlated significantly with tumor grade (*P* < .001), histological type (*P* < .001), age (*P* < .001), KPS (*P* = .0260), tumor status (*P* < .001), and vital status (*P* < .001). Despite the lack of significant differences, a correlated trend was observed for *IDH1* mutation (*P* = .114) and family history of cancer (*P* = .219).

Univariate analysis using logistic regression revealed that *COPB2* expression (ground on median expression value) was linked to poor prognostic clinicopathologic variables (Table 2). Increased *COPB2* expression in glioma was significantly associated with age (≥52 vs <52, OR = 2.35, 95%CI [1.71‐3.23], *P* < .001), vital status (dead vs alive, OR = 2.89, 95%CI [2.07‐4.07], *P* < .001), grade (III vs II, OR = 1.92, 95%CI [1.35‐2.76], *P* < .001; IV vs II, OR = 8.00, 95%CI [5.07‐12.9], *P* < .001), histology type (GBM vs astrocytoma, OR = 4.20, 95%CI [2.70‐6.61], *P* < .001; GBM vs oligoastrocytoma, OR = 6.08, 95%CI [3.67‐10.3], *P* < .001; and GBM vs oligodendroglioma, OR = 5.54, 95%CI [3.52‐8.85], *P* < .001), tumor status (with tumor vs tumor‐free, OR = 3.11, 95%CI [2.17‐4.50], *P* < .001), and KPS (<80 vs ≥80, OR = 1.80, 95%CI [1.07‐3.07], *P* = .03). No significant differences were found on the sex, *IDH1* mutation, ethnicity, and family history of cancer subgroups. These results essentially indicate that *COPB2* may serve as an oncogene and glioma patients with high *COPB2* expression are liable to progress to a more advanced WHO grades, worse histology types, and gain lower KPS points.

**Table 2:** *COPB2* expression associated with clinical‐pathological characteristics (logistic regression)

Clinical characteristics	Total (N)	Odds ratio in *COPB2* expression	*P*‐value
Age (≥52 vs <52)	670	2.35 (1.71‐3.23)	<.001
Sex (male vs female)	670	1.10 (0.81‐1.50)	.530
Vital status (dead vs alive)	670	2.89 (2.07‐4.07)	<.001
Grade (III vs II)	509	1.92 (1.35‐2.76)	<.001
(IV vs II)	409	8.00 (5.07‐12.89)	<.001
Histological type			
(GBM vs astrocytoma)	352	4.20 (2.70‐6.61)	<.001
(GBM vs oligoastrocytoma)	288	6.08 (3.67‐10.29)	<.001
(GBM vs oligodendroglioma)	350	5.54 (3.52‐8.85)	<.001
Tumor status (with tumor vs tumor‐free)	591	3.11 (2.17‐4.50)	<.001
*IDH1* mutation (yes vs no)	126	0.510 (0.220‐1.12)	.100
KPS (<80 vs ≥80)	413	1.80 (1.07‐3.07)	.0300
Ethnicity (Hispanic or Latino vs not Hispanic or Latino)	609	1.28 (0.640‐2.61)	.490
Family history of cancer (yes vs no)	338	1.22 (0.790‐1.89)	.370

### 3.4 Survival outcomes and multivariate analysis

To investigate the predictive implications of *COPB2* in glioma prognosis, we analyzed *COPB2* expression and the OS in the TCGA database. After eliminating patients with absent OS data, remaining patients underwent a Kaplan‐Meier analysis. Glioma patients with *COPB2*‐high had a worse prognosis than that with *COPB2*‐low (Figure 2I, *P* < .001).

Univariate and multivariate Cox analyses were conducted to further explore the prognostic value of *COPB2*. In total, 342 gliomas patients with integrated data containing all the variables were analyzed. The univariate Cox regression revealed that *COPB2*‐high correlated significantly with a worse OS (hazard ratio [HR]: 1.13, 95%CI [1.09‐1.18], *P* < .001) (Table 3). Other clinicopathologic characteristics associated with poor survival were age (HR = 3.70, 95%CI [2.52‐5.44]), WHO grade (HR = 5.32, 95%CI [3.88‐7.31]), histological type (HR = 1.89, 95%CI [1.54‐2.30]), KPS (HR = 2.26, 95%CI [1.41‐3.62]), and tumor status (HR = 39.7, 95%CI [5.53‐284]), all with *P* < .001. The multivariate Cox analysis identified *COPB2* remained independently associated with OS, with a HR of 1.05 (CI: 1.01‐1.08, *P* = .006), along with tumor status.

**Table 3:** (A) Univariate analysis of clinicopathologic characteristics and overall survival in The Cancer Genome Atlas cohort. (B) Multivariate analysis postvariable selection

Characteristics	Hazard ratio (95% CI)	*P*‐value
A.		
Age	3.70 (2.52‐5.44)	<.001
Gender	1.08 (0.750‐1.55)	.696
Grade	5.32 (3.88‐7.31)	<.001
Histological type	1.89 (1.54‐2.30)	<.001
KPS	2.26 (1.41‐3.62)	<.001
Tumor status	39.7 (5.53‐284)	<.001
Ethnicity	0.480 (0.150‐1.51)	.209
*COPB2* expression	1.13 (1.09‐1.18)	<.001
B.		
Tumor status	4.51 (3.29‐6.18)	<.001
Histological type	1.07 (0.900‐1.27)	.452
*COPB2* expression	1.05 (1.01‐1.08)	.00600

### 3.5 *COPB2*‐related signaling pathways based on GSEA

As many signaling pathways contribute to tumor initiation and progression, the poor prognosis of *COPB2*‐high may be related to the numerous signaling pathways activated in glioma. GSEA was utilized to recognize signaling pathways involved in glioma between low and high *COPB2* expression cohorts. Significant differences (normalized *P* < .05, FDR < 0.05) were observed in the enrichment of the MSigDB Collection (kegg.v6.2.symbols.gmt).

**Figure 3:**
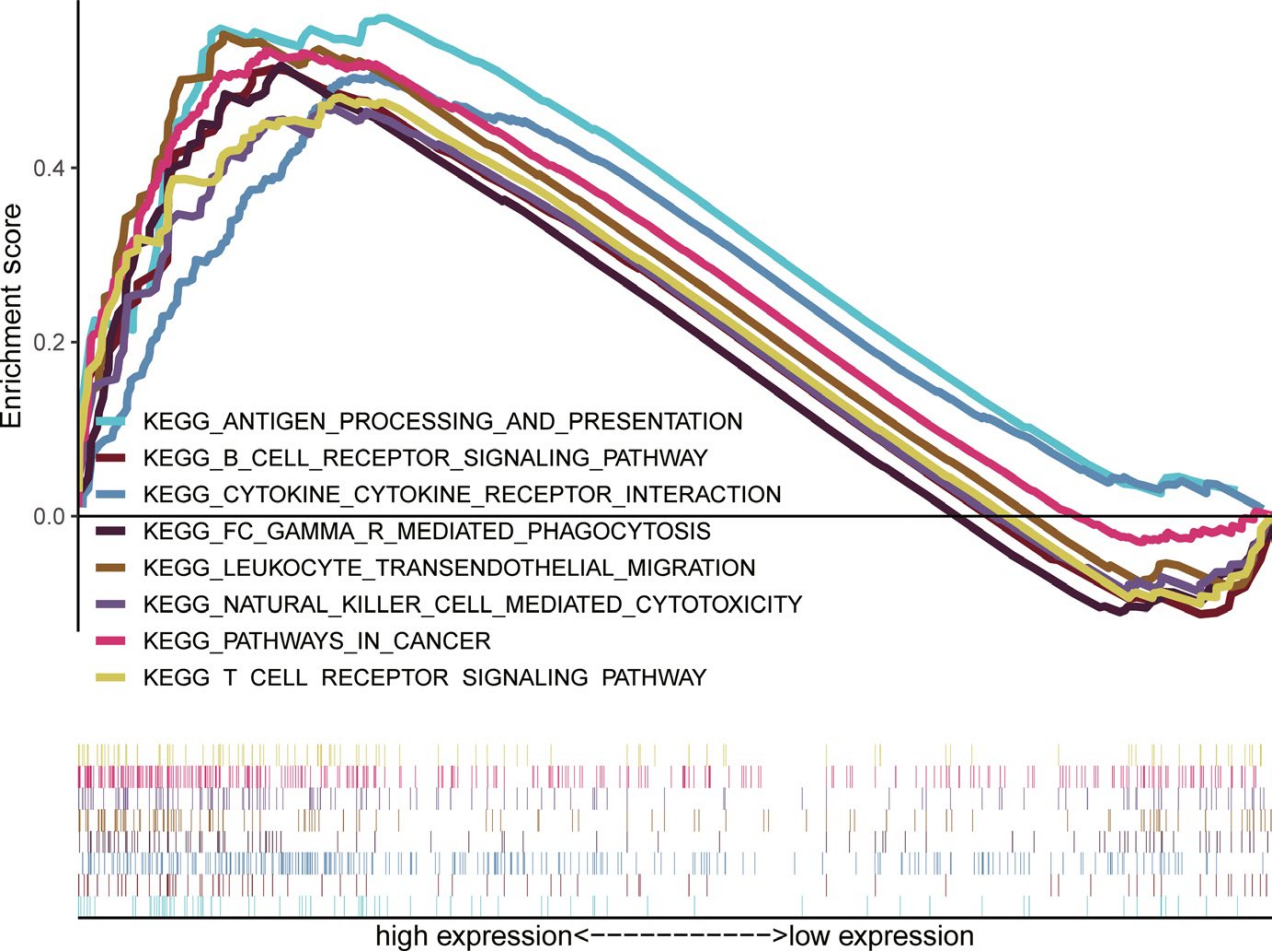
Enrichment plots from gene set enrichment analysis (GSEA)

Several signaling pathways—especially inflammation‐ and immunity‐related pathways—were enriched in the *COPB2* high expression phenotype, including B‐cell receptor, T‐cell receptor, natural killer (NK) cell‐mediated cytotoxicity, antigen processing and presentation, Fc gamma R‐mediated phagocytosis, cytokine‐cytokine receptor interaction, leukocyte transendothelial migration, and other pathways in cancer (please see Figure 3 and Table 4).

**Table 4:** Gene sets enriched in high *COPB2* expression phenotype

MSigDB collection	Gene set name	NES	NOM *P*‐value	FDR *q*‐value
Kegg.v6.2.symbols.gmt	KEGG_ANTIGEN_PROCESSING_AND_PRESENTATION	1.78	.0180	0.0250
	KEGG_B_CELL_RECEPTOR_SIGNALING_PATHWAY	1.73	.0240	0.0300
	KEGG_T_CELL_RECEPTOR_SIGNALING_PATHWAY	1.68	.0250	0.0390
	KEGG_FC_GAMMA_R_MEDIATED_PHAGOCYTOSIS	1.80	.00800	0.0220
	KEGG_NATURAL_KILLER_CELL_MEDIATED_CYTOTOXICITY	1.66	.0330	0.0420
	KEGG_LEUKOCYTE_TRANSENDOTHELIAL_MIGRATION	1.91	.00200	0.0110
	KEGG_PATHWAYS_IN_CANCER	2.14	0	0.00300
	KEGG_CYTOKINE_CYTOKINE_RECEPTOR_INTERACTION	1.73	.0270	0.0300

Note

Gene sets with NOM *P*‐value <.05 and FDR *q*‐value <0.05 were considered as significantly enriched.

Abbreviations: FDR, false discovery rate; NES, normalized enrichment score; NOM, nominal.

### 3.6 *COPB2*‐related inflammatory response

**Figure 4:**
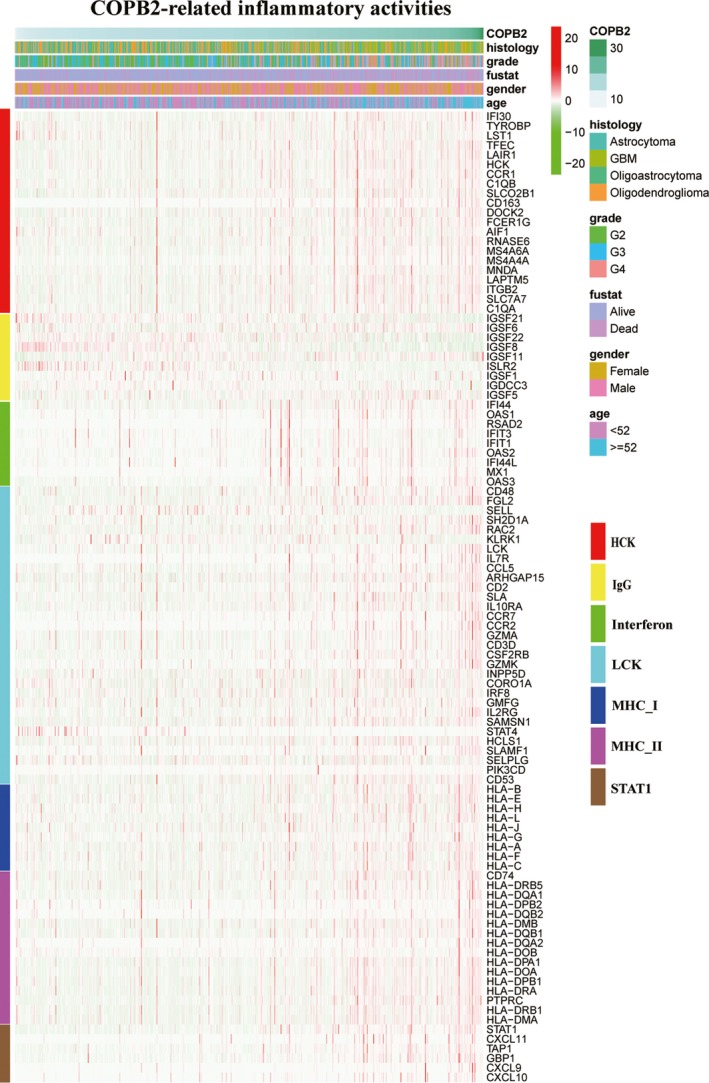
COPB2‐related inflammatory response. Heat maps displaying COPB2 expression, the clinicopathological parameters, and seven well‐established metagenes from the TCGA datasets

**Figure 5:**
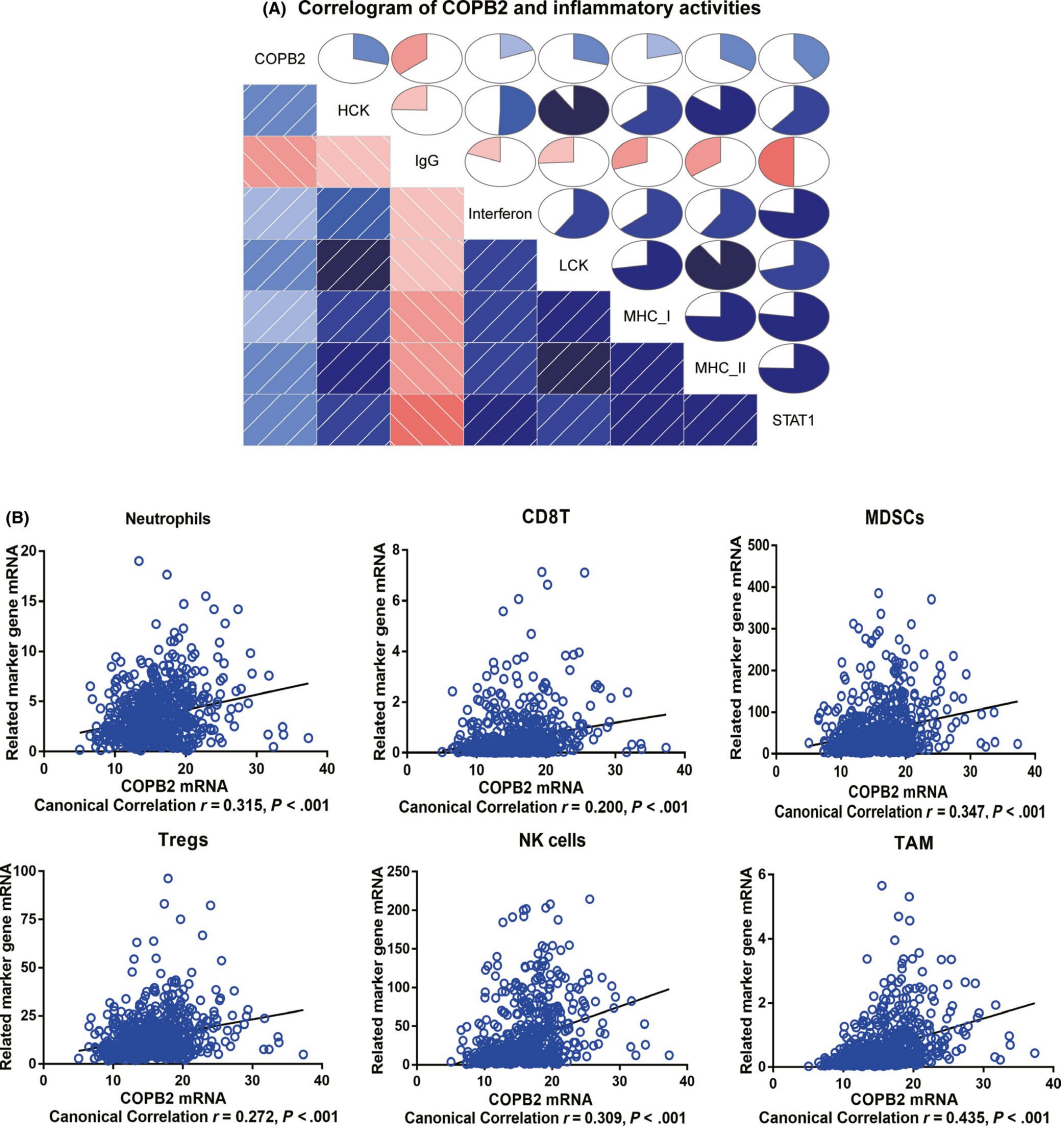
A, Correlograms were established based on GSVA enrichment scores for the seven metagenes and COPB2 expression. The circles filled clockwise in blue represented positive values and anticlockwise in red represented negative values. The color depth increased with the absolute values of the correlation. B, Correlations of COPB2 mRNA with immune cell markers. Each circle represented a single sample

To better comprehend *COPB2*‐related inflammatory activities, seven immune system‐related metagene clusters (comprising 104 genes)^14^ that serve as surrogate markers of different immunological cell types were employed (Data S1). *COPB2* expression, metagenes expression, age, gender, vital status, tumor grade, and histology of samples were displayed on a heat map in Figure 4. *COPB2* expression was positively correlated with most genes of the gene sets *HCK, LCK, interferon, STAT1, MHC I,* and *MHC II*, but negatively with most *IgG* genes. To validate the heatmap analysis, a GSVA was conducted to transform gene expression values into enrichment scores for each metagene set. Correlograms were generated (using the R language program) to measure correlations between *COPB2* expression and the enrichment scores of the seven metagenes—these confirmed the aforementioned results (Figure 5A).

### 3.7 Relationship between *COPB2* and infiltrating immune cells

Previous studies reported that tumor‐infiltrating immune cells may represent a crucial pathophysiological factor in the onset and progression of glioma.^15^ We examined the relationship between *COPB2* and six immune cell types that frequently infiltrate the tumor microenvironment, including innate immune cells: neutrophils, tumor‐associated macrophages (TAMs), NK cells, and myeloid‐derived suppressor cells (MDSCs)^16^, ^17^ and adaptive immune cells: CD8+T cells and regulatory T cells (Tregs).^18^, ^19^ The specific biomarker genes of each immune cell type are displayed in Data S2. Canonical correlation analysis revealed that the transcript levels of *COPB2* were positively correlated with the marker gene expression of these six immune cell types in the TCGA datasets (Figure 5B, *P* < .001), indicating that glioma patients with *COPB2*‐high were prone to have more immune cells infiltrated than glioma patients with *COPB2*‐low.

## 4 DISCUSSION

In our study, the quantitative results indicated that *COPB2* had higher expression levels in most cancers compared with normal tissues in the Oncomine database. In addition, *COPB2*’s high expression in patients with glioma was further validated in the TCGA and GEO databases. RNA sequencing data from TCGA were also obtained and analyzed. *COPB2* expression levels were correlated with advanced clinicopathological parameters (high grade, histological type, tumor status, age, vital status, and KPS) and a shorter survival time. Univariate and multivariate Cox analyses showed that *COPB2* may be a promising biomarker for glioma prognosis while a GSEA using TCGA data revealed that some inflammation‐ and immunity‐related pathways and other signaling pathways in cancer are differentially enriched in the *COPB2* high expression phenotype. To further investigate *COPB2*‐related inflammatory activities, heatmaps using seven immune‐related metagenes demonstrated that *COPB2* transcript levels were positively correlated with gene sets for *HCK, LCK, interferon, STAT1, MHC I,* and *MHC II* but negatively with *IgG*, a marker basically associated with B cells. These results were verified by correlograms analysis using GSVA. Moreover, a positive correlation was observed between *COPB2* expression and marker gene expression of all six immune cell types including innate (neutrophils, TAMs, MDSCs, and NK cells) and adaptive (CD8+T cells and Tregs) immune cells by canonical correlation analyses. These results suggested an essential role for *COPB2* in the immune microenvironment of gliomas.

In the last decade, sequencing analysis has been applied comprehensively to explore the molecular mechanisms implicated in the process of disease progression.^20^ Recently, some reports found significant differences in *COPB2*’s expression in various tumors. Underexpression of *COPB2* could downregulate the EMT‐related protein N‐cadherin and vimentin which may promote breast tumor cell invasion.^6^ Knockdown of *COPB2* led to cell apoptosis by inhibiting the RTK signaling cascade molecules in gastric cancer.^21^ In lung adenocarcinoma,^7^ patients with *COPB2*‐high had worse survival status than *COPB2*‐low. In the present study, we demonstrated that the overexpression of *COPB2* in glioma was correlated with advanced clinicopathologic characteristics and predicts worse outcomes. These findings indicated that *COPB2* may be regarded as a promising target for cancer gene therapy.

Glioma cells could release multiple cytokines that promote the infiltration of various immune cells such as MDSCs, microglia, Tregs, macrophages, CD8 T cells, and CD4 T cells into the tumor microenvironment,^22^ as tumors not only recruit immune cells, but also transform said cells into phenotypes that can help tumor cells evade immune system surveillance (Table 5). For example, Roesch et al^23^ reported that macrophages/glioma‐associated microglia (GAMs) tend to gather in tumor sites and generate an immunosuppressive tumor microenvironment which promote glioma invasion, growth, and angiogenesis.^22^, ^24^ Interestingly, one novel discovery of the present study is that *COPB2* was involved in the immune microenvironment of glioma. Here, we observed a correlation between *COPB2* transcript levels and immunosuppressive cell types—such as Tregs, neutrophils, and MDSCs (Figure 5B)—which might exhibit immunosuppressive activities and leading to adverse clinical outcomes in patients with cancer. However, future laboratory research and clinical trials are required to exhaustively explore the interaction between *COPB2* and immunosuppressive cells.

**Table 5:** Secreted and membrane immunosuppressive molecules expressed by glioma cells

Cytokines	Type	Function	References
*TGFβ* *IL‐10* *PGE2* *Gangliosides*	Soluble	Suppressing T‐cell activation, proliferation and differentiation into effector cells.	30, 31
*CD70*	Membrane proteins	Inducing apoptosis of T and B cells from PBMCs.	32
*FASL*		Inducing apoptosis of FAS‐expressing T cells.	33
*HLA‐G*		Inhibiting proliferation, cytotoxicity by interaction with inhibitory receptors expressed on effector lymphocytes.	34
*IDO*		Inhibiting T‐cell proliferation.	35
*PD‐L1*		*PD‐L1* expressed by glioma inhibits *IFN‐γ* production of antitumor T cells.	36

In recent years, the use of immune checkpoint inhibitors (ICIs) has been popular in the treatment of malignant tumors.^25^, ^26^ Some ICIs, such as anti‐*PD‐1* antibody, are superior to traditional chemoradiotherapy in terms of its clinical curative effect.^27^ However, certain types of malignant tumors, such as glioblastoma, are resistant to monotherapy with ICIs.^27^, ^28^ Therefore, new immunotherapeutic targets are urgently needed to treat glioma. Bullock BL et al^29^ demonstrated that *IFN‐γ* signaling represents an important pathway in the resistance to anti–*PD‐1* treatment in cancer. In the present study, *COPB2* expression was observed to be positively associated with the interferon gene sets (Figures 4 and 5A). Thus, it makes sense to try to combine anti–*PD‐1* therapy and anti‐*COPB2* therapy to amplify the efficacy of treatments typically used in isolation.

Some limitations were present in this study: First, the number of patients incorporated in the univariate and multivariate Cox analyses patients were reduced as many patients had missing integrated data on all variables; second, only a small number of healthy samples were used as controls, so additional studies are needed to balance sample size; and lastly, laboratory studies should be carried out to elucidate the precise mechanisms of *COPB2* overexpression in human glioma and clarify its relationship with poor prognosis and immunomodulation.

## 5 CONCLUSION

As far as we are aware, at present, this is the first study to explore the prognostic value of *COPB2* in patients with glioma. This study revealed that *COPB2* expression was upregulated in glioma samples and was related to adverse outcomes. We also found that *COPB2* may represent an important factor in the immunomodulation of the glioma immune microenvironment. Therefore, taken together, *COPB2* may act as a potential biomarker of prognosis and immunotherapeutic target for glioma.

## CONFLICT OF INTEREST

The authors declare no conflict of interest.
