# Supplementary material for: High expression of COPB2 predicts adverse outcomes: A potential therapeutic target for glioma
Source: CNS Neurosci Ther. 2019 Nov 11;26(3):309–18. doi: 10.1111/cns.13254 (PMC7081167; doi:10.1111/cns.13254)
Supplement: Supplementary file 3 [file CNS-26-309-s003.docx]

**Supplementary table: COPB2 expression in different cancers.**

| **Cancer** | **Cancer subtype** |  | **P-value** | **Fold change** | **Rank (10%)** | **Sample** | **Reference** |
| --- | --- | --- | --- | --- | --- | --- | --- |
| **Bladder** | Infiltrating bladder |  | 3.86E-5 | 2.20 | 10 | 27 | [37] |
| **Brain and CNS Cancer** | Glioblastoma |  | 1.09E-14 | 2.31 | 10 | 34 | [38] |
|  | Glioblastoma |  | 5.75E-8 | 2.37 | 10 | 31 | [39] |
| **Breast** | Ductal Breast Carcinoma |  | 8.97E-6 | 2.10 | 10 | 23 | [40] |
|  | Lobular Breast Carcinoma |  | 2.19E-7 | 2.43 | 10 | 24 | [41] |
|  | Invasive Ductal Breast Carcinoma |  | 9.27E-10 | 3.34 | 10 | 42 |  |
| **Cervical** | Cervical Cancer |  | 1.49E-7 | 2.19 | 10 | 42 | [42] |
| **Esophagus** | Barrett's Esophagus |  | 1.77E-8 | 5.05 | 10 | 43 | [43] |
| **Head and Neck Cancer** | Tongue Squamous Cell Carcinoma |  | 8.86E-11 | 2.26 | 10 | 59 | [44] |
|  | Tongue Squamous Cell Carcinoma |  | 2.14E-9 | 2.65 | 10 | 57 | [45] |
| **Leukemia** | B-Cell Acute Lymphoblastic Leukemia |  | 7.43E-6 | -2.38 | 10 | 25 | [46] |
|  | Chronic Lymphocytic Leukemia |  | 2.28E-28 | -2.01 | 10 | 522 | [47] |
| **Lung** | Squamous Cell Lung Carcinoma |  | 1.51E-11 | 2.21 | 10 | 62 | [44] |
| **Lymphoma** | Anaplastic Large Cell Lymphoma |  | 4.04E-8 | 4.62 | 10 | 26 | [48] |
|  | Unspecified Peripheral T-Cell Lymphoma |  | 1.12E-11 | 3.03 | 10 | 48 |  |
|  | Angioimmunoblastic T-Cell Lymphoma |  | 7.11E-5 | 3.52 | 10 | 26 |  |
| **Other** | Malignant Fibrous Histiocytoma |  | 3.02E-6 | 2.51 | 10 | 24 | [49] |

**REFERENCES**

37. Dyrskjot L, Kruhoffer M, Thykjaer T, et al. Gene expression in the

urinary bladder: a common carcinoma in situ gene expression signature

exists disregarding histopathological classification. *Cancer Res*.

2004;64(11):4040‐4048.

38. Shai R, Shi T, Kremen TJ, et al. Gene expression profiling identifies

molecular subtypes of gliomas. *Oncogene*. 2003;22(31):4918‐4923.

39. Bredel M, Bredel C, Juric D, et al. Functional network analysis reveals

extended gliomagenesis pathway maps and three novel MYC‐interacting

genes in human gliomas. *Cancer Res*. 2005;65(19):8679‐8689.

40. Ma XJ, Dahiya S, Richardson E, et al. Gene expression profiling of

the tumor microenvironment during breast cancer progression.

*Breast Cancer Res*. 2009;11(1):R7.

41. Zhao H, Langerod A, Ji Y, et al. Different gene expression patterns

in invasive lobular and ductal carcinomas of the breast. *Mol Biol Cell*.

2004;15(6):2523‐2536.

42. Pyeon D, Newton MA, Lambert PF, et al. Fundamental differences

in cell cycle deregulation in human papillomavirus‐positive and

human papillomavirus‐negative head/neck and cervical cancers.

*Cancer Res*. 2007;67(10):4605‐4619.

43. Hao Y, Triadafilopoulos G, Sahbaie P, et al. Gene expression profiling

reveals stromal genes expressed in common between

Barrett's esophagus and adenocarcinoma. *Gastroenterology*.

2006;131(3):925‐933.

44. Talbot SG, Estilo C, Maghami E, et al. Gene expression profiling

allows distinction between primary and metastatic squamous cell

carcinomas in the lung. *Cancer Res*. 2005;65(8):3063‐3071.

45. Estilo CL, O‐charoenrat P, Talbot S, et al. Oral tongue cancer gene

expression profiling: Identification of novel potential prognosticators

by oligonucleotide microarray analysis. *BMC Cancer*. 2009;9:11.

46. Maia S, Haining WN, Ansen S, et al. Gene expression profiling identifies

BAX‐delta as a novel tumor antigen in acute lymphoblastic

leukemia. *Cancer Res*. 2005;65(21):10050‐10058.

47. Haferlach T, Kohlmann A, Wieczorek L, et al. Clinical utility of microarray‐

based gene expression profiling in the diagnosis and subclassification

of leukemia: report from the International Microarray Innovations

in Leukemia Study Group. *J Clin Oncol*. 2010;28(15):2529‐2537.

48. Piccaluga PP, Agostinelli C, Califano A, et al. Gene expression analysis

of peripheral T cell lymphoma, unspecified, reveals distinct profiles and

new potential therapeutic targets. *J Clin Invest*. 2007;117(3):823‐834.

49. Detwiller KY, Fernando NT, Segal NH, et al. Analysis of hypoxia‐related

gene expression in sarcomas and effect of hypoxia on RNA interference

of vascular endothelial cell growth factor A. *Cancer Res*.

2005;65(13):5881‐5889.
